# Circulating Collagen Metabolites and the Enhanced Liver Fibrosis (ELF) Score as Fibrosis Markers in Systemic Sclerosis

**DOI:** 10.3389/fphar.2022.805708

**Published:** 2022-02-01

**Authors:** Chen Chen, Lingbiao Wang, Jinfeng Wu, Meijuan Lu, Sen Yang, Wenjing Ye, Ming Guan, Minrui Liang, Hejian Zou

**Affiliations:** ^1^ Department of Rheumatology, Huashan Hospital, Fudan University, Shanghai, China; ^2^ Institute of Rheumatology, Immunology and Allergy, Fudan University, Shanghai, China; ^3^ Department of Dermatology, Huashan Hospital, Fudan University, Shanghai, China; ^4^ Department of Laboratory Medicine, Huashan Hospital, Fudan University, Shanghai, China

**Keywords:** enhanced liver fibrosis (ELF) score, hyaluronic acid, modified rodnan skin score (mRSS), procollagen type iii aminoterminal propeptide, systemic sclerosis (SSc), tissue inhibitor for metalloproteinase (TIMP)

## Abstract

**Background:** Serum fibrosis markers for systemic sclerosis (SSc) remain limited. The Enhanced Liver Fibrosis (ELF) score is a collagen marker set consisting of procollagen type III amino terminal propeptide (PIIINP), tissue inhibitor of metalloproteinases 1 (TIMP-1), and hyaluronic acid (HA). This longitudinal study aimed to examine the performance of the ELF score and its single analytes as surrogate outcome measures of fibrosis in SSc.

**Methods:** Eighty-five SSc patients fulfilling the 2013 ACR/EULAR criteria with the absence of chronic liver diseases were enrolled. Serum PIIINP, TIMP-1, HA, and the ELF score were measured and correlated with clinical variables including the modified Rodnan skin score (mRSS) and interstitial lung disease (ILD). Twenty SSc patients underwent a follow-up serological testing and mRSS evaluation during treatment with immunosuppressants and/or anti-fibrotic drugs.

**Results:** Serum PIIINP, TIMP-1, and ELF score were significantly higher in patients with SSc than in healthy controls [PIIINP: 10.31 (7.83–14.10) vs. 5.61 (4.69–6.30), *p* < .001; TIMP-1: 110.73 (66.21–192.45) vs. 61.81 (48.86–85.24), *p* < .001; ELF: 10.34 (9.91–10.86) vs. 9.68 (9.38–9.99), *p* < .001]. Even higher levels of PIIINP, TIMP-1, and ELF score were found in patients with diffuse cutaneous SSc than those with limited cutaneous SSc. At baseline, both PIIINP and ELF score showed good correlation with mRSS (PIIINP: r = .586, *p* < .001; ELF: r = .482, *p* < .001). Longitudinal analysis showed that change in PIIINP positively correlated with change in mRSS (r = 0.701, *p* = .001), while change in ELF score were not related, in a statistical context, to the change in mRSS (ELF: r = .140, *p* = .555). Serum TIMP-1 was significantly higher in SSc patients with ILD, compared to the matched group of patients without ILD [109.45 (93.05–200.09) vs. 65.50 (40.57–110.73), *p* = 0.007].

**Conclusion:** In patients with SSc, the ELF score well correlates with the extent of skin fibrosis, while serum PIIINP is a sensitive marker for longitudinal changes of skin fibrosis. In the future, circulating collagen metabolites may potentially be used to evaluate therapeutic effects of anti-fibrotic treatments in the disease.

## Introduction

Systemic sclerosis (SSc) is a multisystemic connective tissue disease characterized by progressive fibrosis in the skin, lung and other internal organs. Pathological hallmarks of the disease include autoimmune response, collagen accumulation in affected tissues, and obliterative vasculopathy of the peripheral and visceral vasculature (Allanore et al., 2015).

There is an urgent clinical need to identify serum fibrosis markers in SSc. Ideally, the markers should be able to reflect the extent of fibrosis, run parallel with disease progression or regression, and respond to therapeutic interventions. Prior evidence has shown increased collagen synthesis and decreased collagenase activity in SSc derived fibroblasts (LeRoy, 1974; Stone et al., 1995). Consequently, a number of circulating collagen metabolites have been evaluated as candidate biomarkers for SSc over the past decades. However, at the moment, still no validated ones can be reliably used as surrogate outcome measures in clinical trials or routine medical care.

The Enhanced Liver Fibrosis (ELF) score is a serum marker panel consisting of three collagen metabolites, namely procollagen type III amino terminal propeptide (PIIINP), tissue inhibitor of metalloproteinases 1 (TIMP-1) and hyaluronic acid (HA). The composite index was originally derived from patients with chronic liver disease and shown to be predictive of liver fibrosis (Rosenberg et al., 2004; Parkes et al., 2010; Parkes et al., 2011). PIIINP is the amino terminal peptide released during the synthesis and deposition of type III collagen (Lapiere et al., 1971). TIMP-1 is a specific inhibitor of extracellular matrix (ECM) degradation enzymes (Kikuchi et al., 1997). HA is a large glycosaminoglycan involved in the formation of ECM and maintenance of myofibroblast phenotype (Webber et al., 2009). Serum levels of PIIINP, HA, or TIMP-1 were found to be increased in patients with SSc compared with healthy controls (Scheja et al., 1992; Freitas et al., 1996; Young-Min et al., 2001). The clinical implication of those markers further lied in their correlation with SSc disease activity and organ involvement (Kikuchi et al., 1995; Scheja et al., 2000; Yoshizaki et al., 2008). High levels of PIIINP and HA were also demonstrated as unfavorable predictors for survival in SSc (Scheja et al., 1992; Nagy and Czirják, 2005). More recently, the composite index ELF score was found to be superior to its individual components in reflecting overall fibrotic activity in two independent SSc cohorts (Abignano et al., 2014; Abignano et al., 2018). Besides, a pilot study on IgG4-related disease, another immune-mediated fibrotic disease, revealed that the ELF score could also be an useful indicator of treatment response (Della-Torre et al., 2015). However, evidence on SSc is still in limited amounts and longitudinal data are lacking.

This longitudinal study aimed to examine the performance of the ELF score and its single analytes as surrogate outcome measures of fibrosis in SSc.

## Materials and Methods

### Study Subjects

A total of 85 SSc patients were enrolled in this single-center retrospective study. All patients fulfilled the 2013 ACR/EULAR classification criteria for SSc. Exclusion criteria were 1) chronic hepatitis B and C virus infection; 2) alcoholic liver disease; 3) non-alcoholic fatty liver disease; 4) primary biliary cirrhosis; 5) autoimmune hepatitis; 6) primary sclerosing cholangitis; 7) acute liver injury. Eighty-five age- and gender-matched healthy volunteers were recruited as controls. All SSc patients and controls had normal liver function with no signs of liver cirrhosis on ultrasound assessment. The study was approved by the local ethics committee and informed consent was obtained from all participants.

For patients with SSc, disease duration was calculated from the onset of both Raynaud’s phenomenon (RP) and the first non-RP symptom. Modified Rodnan skin score (mRSS) assessment was performed by a same experienced rheumatologist, blinded to the serological testing. Digital ulcer (DU) and pulmonary arterial hypertension (PAH) were identified based on previous literature (Plastiras et al., 2007; Baron et al., 2014). All patients underwent chest high-resolution computed tomography (HRCT) scan. Interstitial lung disease (ILD) was defined as typical radiologic changes affecting more than 5% of the lung parenchyma; the changes include ground-glass attenuation, reticular opacities, and honeycombing, with or without traction bronchiolectasis or bronchiectasis. Two visual semiquantitative HRCT scores were assessed. The overall HRCT score, based on the method describe by Ichikado and colleagues (Ichikado et al., 2006), was graded according to the types and extent (to the nearest 5%) of parenchymal abnormalities in six lung zones. The maximum fibrosis score (MaxFIB) was exclusively calculated from the zone of maximal lung involvement on a scale of 0–4 based on the percentage of area affected (Goldin et al., 2008). Two independent observers evaluated the images and the results were averaged to get the final HRCT scores.

Serum levels of PIIINP, TIMP-1, and HA were measured and clinical evaluations (including mRSS assessment and HRCT scan) were conducted in all the enrolled patients at baseline. Twenty SSc patients underwent a follow-up serological testing and mRSS evaluation during treatment with immunosuppressants and/or anti-fibrotic drugs. Serum was separated and stored at −80°C. The mRSS assessment and HRCT scan were performed within 3 days of the collection of blood samples.

### Biochemical Measurements

The ELF score is an algorithm based on quantitative serum measurements of PIIINP, HA, and TIMP-1. Serum concentrations of PIIINP and HA were measured using the chemiluminescence immunoassay combined with the magnetic particles (MPCLIA) (Autobio Diagnostics, Zhengzhou, China). The MPCLIA combines the competitive enzyme immunoassay with the chemiluminescence technique. In this two-step assay, antigens (PIIINP or HA derivatives) are first pre-coated onto microtiter wells, then reference standards, specimens, and solution containing anti-PIIINP antibody or HA binding proteins are added, respectively. Then the plate is incubated. During incubation, PIIINP or HA presents in specimens or reference standards compete with precoated antigens for combining with PIIINP antibodies or HA binding proteins. When this step is fully completed, wash the microtiter plate to remove unbound materials. Add enzyme conjugate reagent, which combines with anti-PIIINP antibody or HA binding proteins attached on microtiter wells in the previous step. After a second incubation, remove unbound enzyme conjugates by washing. Add chemiluminescent substrates, measure the relative light unit (RLU) value for each well, construct calibration curves with values obtained from reference standards, calculate the serum concentrations of PIIINP and HA in each specimen through the calibration curves. PIIINP and HA concentrations in specimens are inversely proportional to relative light unit (RLU) values. Serum concentrations of TIMP-1 were determined by the sandwich enzyme-linked immunosorbent assay (ELISA) (Sangon Biotech and Bio Basic, Shanghai, China). The procedures are as follow. Add standard and sample to the microplate that have been pre-coated with anti-TIMP-1 antibody. After incubation, add biotin-conjugated anti-TIMP-1 antibody. It is then combined with horseradish peroxidase-conjugated streptavidin to form an immune complex, then incubated and washed to remove unbound enzyme, and then added to the chromogenic substrate TMB (3,3′,5,5′-Tetramethylbenzidine) to produce a blue color and converted to the final yellow under the action of acid. Finally, the absorbance (OD) value was measured at 450 nm. The concentration of TIMP-1 in the sample was proportional to the absorbance (OD) value and can be calculated by drawing a standard curve. The ELF score was calculated using the Siemens algorithm (Vali et al., 2020):

$$ELF\;score=2.494+0.846\;\ln\left(C_{HA}\right)+0.735\;\ln\left(C_{PIIINP}\right)+0.391\ln\left(C_{TIMP-1}\right)$$

### Statistical Analysis

Statistical analysis was performed using SPSS Statistics (version 28.0), and graphs were constructed using R statistical package (version 4.1.2). Continuous variables were expressed as median (interquartile range) and categorical data as number and percentage. Welch’s t test was used to compare continuous variables between two groups and Mann-Whitney *U* test was applied as an alternative when the parametric assumptions were not met. Chi-square test and when indicated, Fisher’s exact test were used to compare the distribution of categorical variables between groups. Spearman’s rank correlation tests were employed to search for possible relationships between variables and post-hoc power analysis was conducted using Fisher’s asymptotic method. Multiple linear regression was used to evaluate independent predictors of the ELF score. Propensity score matching was used to generate comparable study groups. Propensity scores were estimated by logistic regression and matching was done at 1:1 ratio with a match tolerance of 0.05, based on the maximize execution performance analysis. *p* values of less than .05 (two-sided) were considered statistically significant.

## Results

A total of 85 SSc patients were enrolled, 47 with diffuse cutaneous SSc (dcSSc) and 38 with limited cutaneous SSc (lcSSc). Patient demographic and clinical features are summarized in Table 1. Medium age was 53 (38–62) years, and medium disease duration from the first non-RP symptom was 2.0 (1.0–5.0) years. Patient mRSS ranged from 0 to 41 with a medium of 8 (interquartile range: 3.5–17.5). Patients with DU, ILD, and PAH accounted for 32.9% (28), 57.6% (49), and 4.7% (4) of the study population, respectively. Positive anti-topoisomerase I antibody (ATA), anti-centromere antibody (ACA), and anti-RNA polymerase III antibody (ARA) were present in 47 (55.3%), 19 (22.4%), and 4 (4.7%) patients, respectively.

**TABLE 1:** Demographic and clinical characteristics of 85 SSc patients.

Characteristics	N = 85
Gender (female/male)	68/17 (80.0%/20.0%)
Age, years	53 (38–62)
Subtype (dcSSc/lcSSc)	47/38 (55.3%/44.7%)
Disease duration from RP, years	3.0 (1.0–9.0)
Disease duration from first non-RP, years	2.0 (1.0–5.0)
mRSS	8.0 (3.5–17.5)
Raynaud’s phenomenon	79 (92.9%)
Puffy fingers	58 (68.2%)
Telangiectasia	46 (54.1%)
Digital ulcer	28 (32.9%)
Interstitial lung disease	49 (57.6%)
Pulmonary arterial hypertension	4 (4.7%)
ESR, mm/h	21.0 (12.0–37.0)
Disease specific autoantibodies	
ATA positive	47 (55.3%)
ACA positive	19 (22.4%)
ARA positive	4 (4.7%)

ACA, anti-centromere antibody; ARA, anti-RNA polymerase III antibody; ATA, anti-topoisomerase I antibody; dcSSc, diffuse cutaneous SSc; ESR, erythrocyte sedimentation rate; lcSSc, limited cutaneous SSc; mRSS, modified Rodnan skin score; RP, Raynaud’s phenomenon; SSc, systemic sclerosis.

### Elevated Collagen Metabolites in Patients With SSc

**FIGURE 1:**
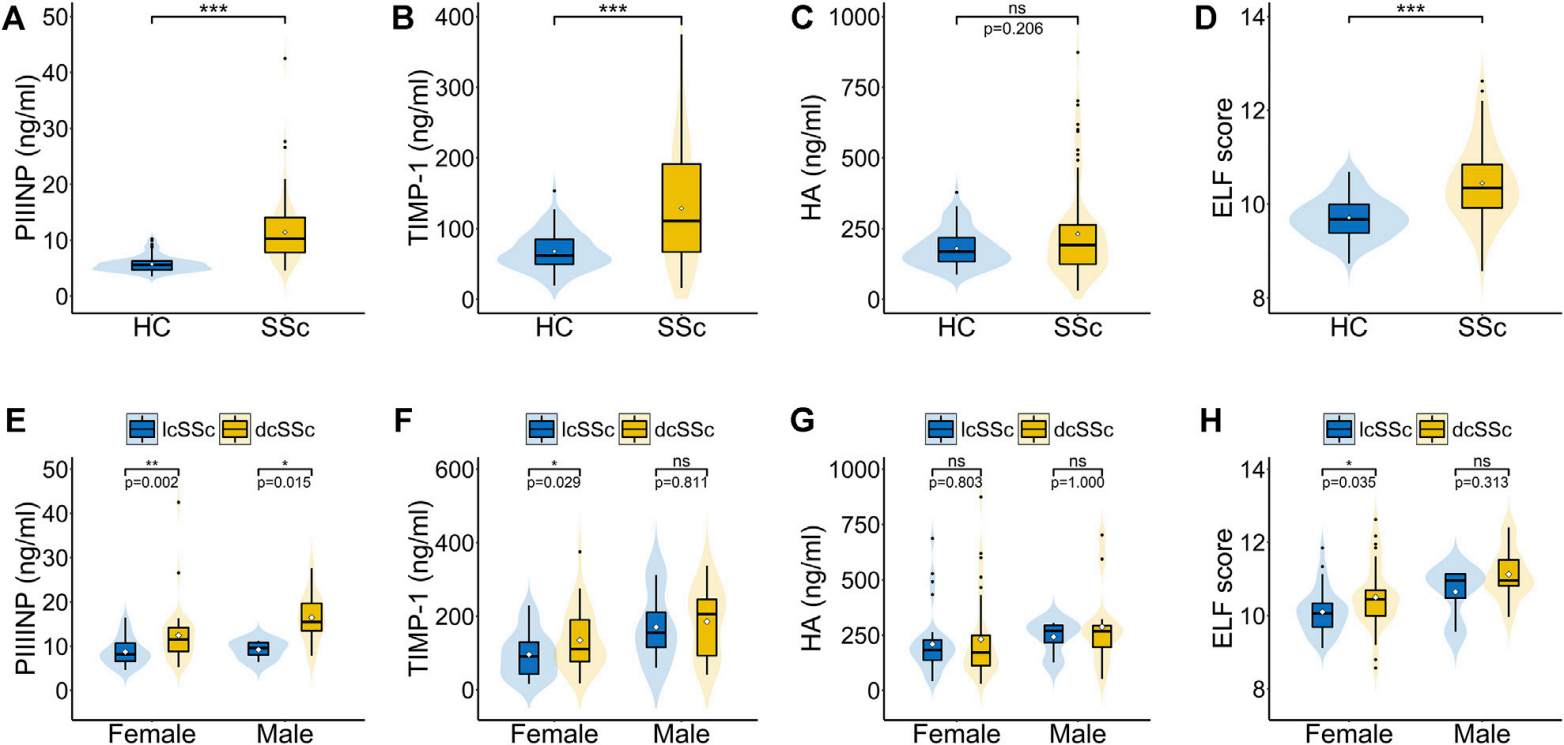
Serum PIIINP, TIMP-1, HA levels and ELF score in patients with SSc. **(A–D)** Serum PIIINP, TIMP-1, HA levels and ELF score in patients with SSc compared with healthy controls. **(E–H)** Serum PIIINP, TIMP-1, HA levels and ELF score in patients with diffuse cutaneous SSc compared with limited cutaneous SSc in males and females. A violin plot includes a fat line showing the median of the data, a box representing the interquartile range, upper and lower bars indicating the 5th and 95th centiles, and a shadow presenting the kernel probability density of the data at different values. The black dots stand for outliers and the white diamonds stand for mean values. HC, healthy controls; dcSSc, diffuse cutaneous SSc; lcSSc, limited cutaneous SSc; ns, not significant. ***p < .001, **p < .01, *p < .05.

Serum PIIINP, TIMP-1, and ELF score were significantly higher in patients with SSc than in healthy controls [PIIINP: 10.31 (7.83–14.10) vs. 5.61 (4.69–6.30), *p* < .001; TIMP-1: 110.73 (66.21–192.45) vs. 61.81 (48.86–85.24), *p* < .001; ELF: 10.34 (9.91–10.86) vs. 9.68 (9.38–9.99), *p* < .001], while no statistically significant difference was observed in serum HA between SSc patients and healthy controls [192.00 (124.17–265.07) vs. 168.16 (132.57–219.11) *p* = .206; Figures 1A–D]. Among patients, males showed remarkably higher PIIINP, TIMP-1, HA, and ELF score than females (*p* = .003, *p* = .008, *p* = .020, *p* = .001, respectively; Table 2), whereas no such gender dependency could be observed in healthy controls (*p* = .525, *p* = .160, *p* = .273, *p* = .257, respectively). Even higher levels of PIIINP, TIMP-1, and ELF score were found in dcSSc compared with lcSSc (*p* < .001, *p* = .017, *p* = .003, respectively; Table 2). Since there was a higher percentage of males in the dcSSc group than in the lcSSc group (27.7% vs. 10.5%, *p* = .050), a subgroup analysis by gender was further conducted to eliminate possible confounding. The results demonstrated still higher levels of PIIINP, TIMP-1, and ELF score in dcSSc in the female subgroup (*p* = .002, *p* = .029, *p* = 0.035, respectively; Figures 1E–H).

**TABLE 2:** Serum PIIINP, TIMP-1, HA levels and ELF score in patients with SSc stratified by main clinical characteristics.

Characteristics	PIIINP (ng/ml)	TIMP-1 (ng/ml)	HA (ng/ml)	ELF score
Total	10.31 (7.83–14.10)	110.73 (66.21–192.45)	192.00 (124.17–265.07)	10.34 (9.91–10.86)
Gender				
female	9.50 (7.14–13.22)	104.90 (63.88–166.75)	178.33 (117.40–243.53)	10.23 (9.79–10.64)
male	14.05 (9.80–19.50)	197.73 (90.94–256.83)	266.97 (166.52–299.14)	10.96 (10.79–11.47)
*p* Value	0.003	0.008	0.020	0.001
Subtype				
dcSSc	13.34 (8.99–15.46)	116.00 (85.18–214.77)	195.88 (121.84–275.86)	10.60 (10.11–11.20)
lcSSc	8.39 (6.55–10.91)	98.32 (48.50–141.75)	188.50 (133.90–246.52)	10.10 (9.65–10.53)
*p* Value	<0.001	0.017	0.691	0.003
DU				
with DU	10.91 (8.62–14.18)	118.35 (70.89–199.50)	171.58 (124.17–302.18)	10.38 (9.98–11.10)
w/o DU	9.65 (7.23–14.10)	107.12 (53.06–185.34)	202.09 (121.87–255.24)	10.31 (9.84–10.81)
*p* Value	0.282	0.175	0.874	0.364
ILD				
with ILD	10.90 (7.99–14.06)	120.70 (80.00–206.49)	202.82 (113.63–273.63)	10.37 (9.95–11.05)
w/o ILD	9.61 (7.51–14.90)	95.83 (51.76–166.75)	179.83 (133.87–253.16)	10.24 (9.82–10.83)
*p* Value	0.715	0.054	0.803	0.600
PAH				
with PAH	10.99 (9.07–14.56)	92.81 (31.70–212.59)	172.34 (132.98–370.11)	10.24 (10.04–11.08)
w/o PAH	10.44 (7.83–14.13)	110.73 (66.93–193.66)	195.88 (123.77–266.97)	10.40 (9.91–10.88)
*p* Value	0.659	0.674	0.926	1.000
ATA				
ATA+	10.97 (7.97–14.42)	120.70 (85.18–205.80)	202.09 (121.84–266.97)	10.32 (9.91–11.01)
ATA-	9.61 (7.72–13.56)	99.76 (62.59–170.03)	178.33 (126.30–267.29)	10.39 (9.89–10.84)
*p* Value	0.331	0.110	0.757	0.965
ACA				
ACA+	8.59 (7.41–10.97)	65.50 (34.99–120.94)	178.26 (123.77–228.01)	10.20 (9.79–10.56)
ACA-	10.94 (7.93–14.27)	118.35 (82.58–202.52)	199.26 (123.89–273.50)	10.40 (9.94–11.13)
*p* Value	0.143	0.003	0.776	0.187

ACA, anti-centromere antibody; ARA, anti-RNA polymerase III antibody; ATA, anti-topoisomerase I antibody; dcSSc, diffuse cutaneous SSc; DU, digital ulcer; ELF, enhanced liver fibrosis; HA, hyaluronic acid; ILD, interstitial lung disease; lcSSc, limited cutaneous SSc; PAH, pulmonary arterial hypertension; PIIINP, procollagen type III amino terminal propeptide; SSc, systemic sclerosis; TIMP-1, tissue inhibitor of metalloproteinases 1; w/o, without.

As shown in Table 3, serum PIIINP, HA, and ELF score negatively correlated with disease duration from RP (PIIINP: r = −.348, *p* = .001, HA: r = −.320, *p* = .003; ELF: r = −.400, *p* < .001). Serum HA and ELF score positively correlated with age (HA: r = .456, *p* < .001, ELF: r = .353, *p* = .001). A similar age-related trend was also found in healthy controls (HA: r = .364, *p* = .001, ELF: r = .284, *p* = .012).

**TABLE 3:** Correlation coefficient (r) between PIIINP, TIMP-1, HA, ELF score and continuous clinical variables in patients with SSc.

Characteristics	PIIINP	TIMP-1	HA	ELF score
Age	−0.001	0.001	0.456^***^	0.353^**^
Disease duration from RP	−0.348^**^	−0.175	−0.320^**^	−0.400^***^
Disease duration from first non-RP	−0.226^*^	−0.134	−0.197	−0.264^*^
mRSS	0.586^***^	0.240^*^	0.237^*^	0.482^***^
Overall HRCT score^a^	−0.152	0.100	0.280	0.255
MaxFIB HRCT score^a^	−0.095	−0.001	0.189	0.158
ESR, mm/h	0.263^*^	0.105	0.086	0.215

ELF, enhanced liver fibrosis; ESR, erythrocyte sedimentation rate; HA, hyaluronic acid; HRCT, high resolution computed tomography; MaxFIB, maximum fibrosis score; mRSS, modified Rodnan skin score; PIIINP, procollagen type III amino terminal propeptide; RP, Raynaud’s phenomenon; SSc, systemic sclerosis; TIMP-1, tissue inhibitor of metalloproteinases 1.

a Only patients with interstitial lung disease (N = 49) were included in the analysis.

***p < .001.

**p < .01.

*p < .05.

### Collagen Metabolites as Indicators of Skin Fibrosis

**FIGURE 2:**
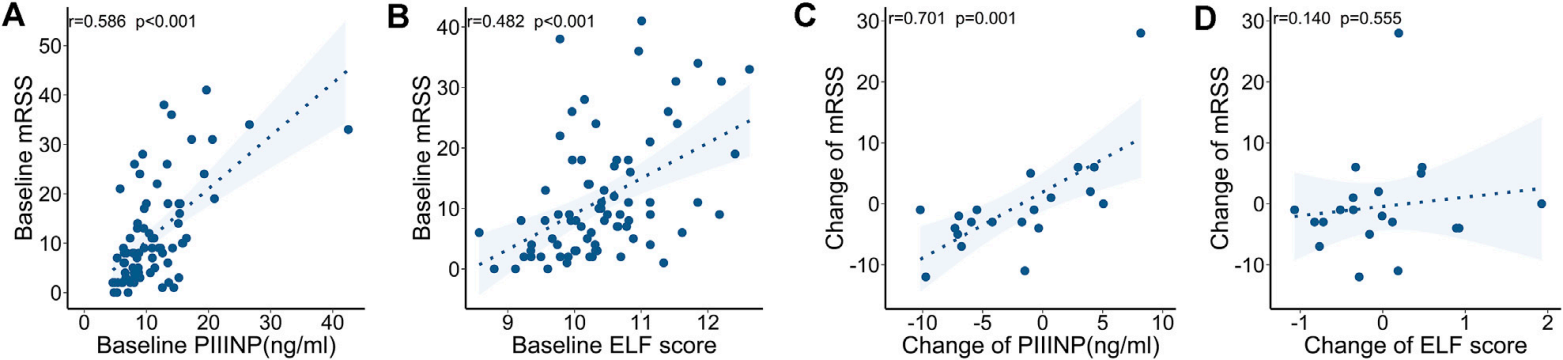
Correlations between serum collagen markers and mRSS at baseline and during follow-up. **(A)** Serum PIIINP and mRSS at baseline. **(B)** ELF score and mRSS at baseline. **(C)** Change in PIIINP and change in mRSS over time. **(D)** Change in ELF score and change in mRSS over time.

At baseline, both PIIINP and ELF score showed strong correlation with mRSS, while only moderate correlations were observed for TIMP-1 or HA (PIIINP: r = .586, *p* < .001, power = .999; TIMP-1: .240, *p* = .035, HA: r = .237, *p* = .038; ELF: r = .482, *p* < .001; Table 3 and Figures 2A,B). Multiple linear regression analysis was performed and all the variables that were significantly correlated with those serum collagen metabolites in the univariate analysis were included as covariates. In multiple linear regression models, mRSS maintained to be a strong predictor for serum PIIINP, HA, and ELF score (p<.001, p = .012, p<.001, respectively).

The follow-up mRSS evaluation and serological testing were made at a medium of 3.4 (interquartile range: 2.1–9.6) months after the baseline assessment in 20 SSc patients. The clinical features and ongoing medications are summarized in Table 4. Specifically, immunosuppressants (including cyclophosphamide, mycophenolate mofetil, and methotrexate) and anti-fibrotic drugs (including nintedanib and pirfenidone) were used in 10 (50%) and 9 (45%) patients, respectively. In a longitudinal view, changes in PIIINP positively correlated with changes in mRSS (r = .701, *p* = .001, power = .895); in most cases, decrease in serum PIIINP levels was in parallel with improvement in skin fibrosis (Figure 2C). No significant correlations were observed between changes in TIMP-1, HA, or ELF and changes in mRSS (TIMP-1: r = −.170, *p* = .474; HA: r = −.126, *p* = .596, ELF: r = .140, *p* = .555; Figure 2D).

**TABLE 4:** Clinical features and ongoing medications of the 20 SSc patients with longitudinal follow-ups.

Characteristics	N = 20
Follow-up time, months	3.4 (2.1–9.6)
Gender (female/male)	14/6 (70%/30%)
Age, years	56 (50–62)
Subtype (dcSSc/lcSSc)	14/6 (70%/30%)
Disease duration from RP, years	1.7 (0.5–6.0)
Disease duration from first non-RP, years	1.7 (0.6–3.0)
mRSS	9.5 (7.0–21.8)
Interstitial lung disease	14 (70%)
ESR, mm/h	27.0 (19.0–37.0)
Disease specific autoantibodies	
ATA positive	17 (85%)
ACA positive	1 (5%)
ARA positive	1 (5%)
Treatment	
Immunosuppressants^a^	10 (50%)
Anti-fibrotic drugs^b^	9 (45%)

ACA, anti-centromere antibody; ARA, anti-RNA polymerase III antibody; ATA, anti-topoisomerase I antibody; dcSSc, diffuse cutaneous SSc; ESR, erythrocyte sedimentation rate; lcSSc, limited cutaneous SSc; mRSS, modified Rodnan skin score; RP, Raynaud’s phenomenon.

a Immunosuppressants include cyclophosphamide, mycophenolate mofetil, and methotrexate.

b Anti-fibrotic drugs include nintedanib and pirfenidone.

### Collagen Metabolites as Markers of Lung Fibrosis

Neither ELF nor its single components showed statistically significant differences between patients with or without ILD, although trends toward higher serum levels of collagen metabolites in patients with ILD were observed (Table 2). No linear correlation between collagen markers and semi-quantitative HRCT scores could be established either (Table 3).

To balance the distribution of demographic and clinical features, SSc patients with and without ILD were matched for age, gender, disease subtype, disease duration from RP onset and mRSS using propensity score matching. After matching, 30 patients remained (15 in each group) and two groups were well balanced in terms of the matched variables (Table 5). Serum TIMP-1 was significantly higher in patients with ILD, compared to the matched group of patients without ILD [109.45 (93.05–200.09) vs. 65.50 (40.57–110.73), *p* = .007], while no statistical difference was found in PIIINP, HA, and ELF score (*p* = .412, *p* = .285, *p* = .215, respectively; Table 5).

**TABLE 5:** Clinical and serological parameters of SSc patients with and without ILD matched by propensity score matching.

Characteristics	With ILD (*n* = 15)	Without ILD (*n* = 15)	SMD	*p* Value
Age, years	54 (41–62)	56 (44–64)	0.149	0.686
Gender (female/male)	12/3 (80%/20%)	14/1 (93.3%/6.7%)	0.400	0.598
Subtype (dcSSc/lcSSc)	10/5 (67%/33%)	10/5 (67%/33%)	<0.001	1.000
Disease duration from RP, years	3.0 (1.7–10.0)	4.0 (1.5–10.0)	0.126	0.967
mRSS	11.0 (6.0–24.0)	10 (3.0–14.0)	0.392	0.367
Overall HRCT score	145 (125–159)	—	—	—
MaxFIB HRCT score	3 (2–3)	—	—	—
PIIINP, ng/ml	9.66 (6.59–12.02)	12.29 (7.83–15.23)	0.318	0.412
TIMP-1, ng/ml	109.45 (93.05–200.09)	65.50 (40.57–110.73)	0.908	0.007
HA, ng/ml	228.14 (125.37–273.37)	178.26 (92.93–231.85)	0.090	0.285
ELF score	10.20 (9.89–10.68)	10.40 (10.15–11.13)	0.463	0.215

ELF, enhanced liver fibrosis; HA, hyaluronic acid; HRCT, high resolution computed tomography; ILD, interstitial lung disease; MaxFIB, maximum fibrosis score; mRSS, modified Rodnan skin score; PIIINP, procollagen type III amino terminal propeptide; RP, Raynaud’s phenomenon; SMD, standardized mean difference; TIMP-1, tissue inhibitor of metalloproteinases 1.

## Discussion

The present study illustrates longitudinal changes of serum collagen metabolites in SSc. A novel finding is the strong positive correlation between changes of PIIINP and changes of mRSS over time, which reveals that PIIINP could serve as a potent biomarker for monitoring skin sclerosis and active fibrosis in SSc.

Previous reports have documented a remarkable decrease in serum PIIINP after plasma exchange or immunosuppressive therapy in patients with SSc (Behr et al., 1995; Heickendorff et al., 1995; Cozzi et al., 2001). However, the clinical relevance of the serological changes remains elusive. In order to address this issue, we concurrently assess the serum collagen metabolites and the clinical parameter mRSS. Our data suggested that serum PIIINP could be a surrogate outcome measure in SSc, in parallel with the skin score. However, it should be noted that changes in serum PIIINP could not be linked to any specific treatment regimen in our study, since patients received various types of therapy, including immunosuppressants and anti-fibrotic drugs, either alone or in combination. Future studies are needed to investigate the potential of those collagen markers in monitoring therapeutic efficacy in different treatment subgroups of SSc patients.

Interestingly, longitudinal change in PIIINP exhibited strong correlation with longitudinal change in mRSS, probably superior to the ELF score in reflecting skin progression in patients with SSc. This may result from the multiparametric nature of the ELF algorithm, where every single marker reflects a different aspect of the fibrotic process. Indeed, PIIINP and TIMP-1 respectively represent increased collagen formation and decreased collagen degradation. HA was superior in identification of advanced fibrosis but much weaker than PIIINP or TIMP-1 in discriminating early fibrosis (Lichtinghagen et al., 2013). Considering the short disease duration and moderate mRSS of our study population, current data revealed that PIIINP could be a sensitive surrogate marker of fibrotic burden and disease progression in patients with early SSc. It should also be highlighted that the ELF score was initially derived from a chronic liver disease cohort. The algorithm and reference ranges, specially tailored to assess liver fibrosis and predict liver-related events, may not be readily applicable to SSc. Our results reaffirmed the necessity to develop a disease-specific algorithm based on the clinical implication and analytical performance of individual markers in SSc.

It should also be highlighted that every single marker is differentially affected by confounding factors, which can further complicate the results of the composite score ELF. In the present study as well as previous reports, age is established as a putative confounder for serum HA in both healthy controls and SSc patients (Lichtinghagen et al., 2013; Dellavance et al., 2016). Besides, the diet-related intraday variation of serum HA was reported to be as high as 70% (Lichtinghagen et al., 2013). Disease duration is another influencing factor to be reckoned with. Patients with shorter SSc disease course tended to have higher levels of collagen metabolites, which can be attributed to the active ECM turnover in early phase of the disease (Scheja et al., 2000), and is also in agree with the spontaneous regression of skin fibrosis in some SSc patients (Allanore et al., 2015). Hence, we believe that those serum collagen markers are more valuable when used for within-individual comparisons during follow-ups, while between-individual comparisons should be avoided to prevent misinterpretation of the results.

Recently, Dobrota and colleagues reported that high serum levels of PRO-C3 (the PIIINP neo-epitope) significantly predicted skin progression in SSc, after adjustment for mRSS, sex, and age (Dobrota et al., 2021). PRO-C3 is identified as a more dynamic marker of fibrogenesis than PIIINP, since the antibody applied in PRO-C3 assay only recognized the epitope on PIIINP where the propeptide is cleaved from the intact type III collagen (Nielsen et al., 2013). However, considering that previous investigations in healthy controls found no significant correlation between serum levels of PRO-C3 and PIIINP (Nielsen et al., 2013), the clinical implications of those two serum biomarkers still require further comparing and exploring in SSc cohorts. In the current study, an attempted has also been made to evaluate the predictive effects of serum collagen markers on longitudinal change in mRSS. However, the results were inconclusive due to the small sample size and larger samples are required before valid conclusions can be drawn.

In regard to lung involvement, Abignano and colleagues demonstrated that the ELF score was significantly higher in patients with ILD compared to those without (Abignano et al., 2014), while in the present study, only TIMP-1 showed difference between the two groups of patients. The conflicting results may be explained by the fact that the ELF score is more of a marker for overall fibrotic activity in SSc, rather than a marker for specific organ involvement (Abignano et al., 2018). Though propensity score matching was used to balance the distribution of several demographic and clinical features in our study, multisystem fibrotic conditions could still complicate the results. Indeed, it is no easy job to evaluate ILD using serological markers alone, and a combined clinical and collagen marker algorithm to predict lung function decline in SSc is currently under development (Hutchinson et al., 2021).

This study is limited by the sample size and retrospective design. Serial measurements are warranted to determine the sensitivity of serum collagen markers to fibrotic changes over a longer period of time. Second, the minimal clinically important difference of serum biomarkers was not estimated in the study, making it hard to evaluate the effect of a given therapy through the serological measurements. Third, potential serum markers for progression of ILD are also well worthy to be explored in patients with SSc. However, due to the retrospective nature of the study, sequential pulmonary function tests were only performed in less than half of the patients and no conclusion can be drawn from this extremely small cohort at the moment. In addition, there is a technical detail to be mentioned that serum PIIINP, TIMP-1, and HA were analyzed using commercial reagents, rather than the integrated platform for ELF in the current study. Considering the inevitable manufacturer-based variation, cross-study comparisons of data from the present study and previous literature have to be made with care.

In conclusion, our study shed new light on the validity of serum collagen metabolites as reliable markers of fibrosis in SSc. We unveil for the first time that changes in PIIINP correlate well with changes in clinical outcome. In the future, collagen markers could potentially be used to evaluate therapeutic effects of anti-fibrotic treatments in SSc. Research is also needed to establish a disease-specific collagen index to better reflect the fibrotic process in SSc.

## Data Availability

The raw data supporting the conclusion of this article will be made available by the authors, without undue reservation.

## Abbreviations

ACA, anti-centromere antibody; ARA, anti-RNA polymerase III antibody; ATA, anti-topoisomerase I antibody; dcSSc, diffuse cutaneous systemic sclerosis; DU, digital ulcer; ECM, extracellular matrix; ELF, Enhanced Liver Fibrosis; ELISA, enzyme-linked immunosorbent assay; HA, hyaluronic acid; HC, healthy control; HRCT, high-resolution computed tomography; ILD, interstitial lung disease; lcSSc, limited cutaneous systemic sclerosis; MaxFIB, maximum fibrosis score; MPCLIA, chemiluminescence immunoassay combined with the magnetic particles; mRSS, modified Rodnan skin score; PAH, pulmonary arterial hypertension; PIIINP, procollagen type III amino terminal propeptide; RP, Raynaud’s phenomenon; SSc: systemic sclerosis; TIMP-1, tissue inhibitor of metalloproteinases 1.

## References

[B1] Abignano G. Cuomo G. Buch M. H. Rosenberg W. M. Valentini G. Emery P. (2014). The Enhanced Liver Fibrosis Test: a Clinical Grade, Validated Serum Test, Biomarker of Overall Fibrosis in Systemic Sclerosis. Ann. Rheum. Dis. 73, 420–427. 10.1136/annrheumdis-2012-202843 23511226

[B2] Abignano G. Blagojevic J. Bissell L.-A. Dumitru R. B. Eng S. Allanore Y. (2018). European Multicentre Study Validates Enhanced Liver Fibrosis Test as Biomarker of Fibrosis in Systemic Sclerosis. Rheumatology 58, 254. 10.1093/rheumatology/key271 30239834

[B3] Allanore Y. Simms R. Distler O. Trojanowska M. Pope J. Denton C. P. (2015). Systemic Sclerosis. Nat. Rev. Dis. Primers 1, 15002. 10.1038/nrdp.2015.2 27189141

[B4] Baron M. Chung L. Gyger G. Hummers L. Khanna D. Mayes M. D. (2014). Consensus Opinion of a North American Working Group Regarding the Classification of Digital Ulcers in Systemic Sclerosis. Clin. Rheumatol. 33, 207–214. 10.1007/s10067-013-2460-7 24357325

[B5] Behr J. Adelmann-Grill B. C. Hein R. Beinert T. Schwaiblmair M. Krombach F. (1995). Pathogenetic and Clinical Significance of Fibroblast Activation in Scleroderma Lung Disease. Respiration 62, 209–216. 10.1159/000196449 8578017

[B6] Cozzi F. Marson P. Rosada M. De Silvestro G. Bullo A. Punzi L. (2001). Long-term Therapy with Plasma Exchange in Systemic Sclerosis: Effects on Laboratory Markers Reflecting Disease Activity. Transfus. Apher. Sci. 25, 25–31. 10.1016/s1473-0502(01)00078-7 11791759

[B7] Della-Torre E. Feeney E. Deshpande V. Mattoo H. Mahajan V. Kulikova M. (2015). B-cell Depletion Attenuates Serological Biomarkers of Fibrosis and Myofibroblast Activation in IgG4-Related Disease. Ann. Rheum. Dis. 74, 2236–2243. 10.1136/annrheumdis-2014-205799 25143523 PMC4806785

[B8] Dellavance A. Fernandes F. Shimabokuro N. Latini F. Baldo D. Barreto J. A. (2016). Enhanced Liver Fibrosis (ELF) Score: Analytical Performance and Distribution Range in a Large Cohort of Blood Donors. Clin. Chim. Acta 461, 151–155. 10.1016/j.cca.2016.08.006 27520747

[B9] Dobrota R. Jordan S. Juhl P. Maurer B. Wildi L. Bay-Jensen A.-C. (2021). Circulating Collagen Neo-Epitopes and Their Role in the Prediction of Fibrosis in Patients with Systemic Sclerosis: a Multicentre Cohort Study. Lancet Rheumatol. 3, e175–e184. 10.1016/S2665-9913(20)30385-4 38279380

[B10] Freitas J. P. Filipe P. Emerit I. Meunier P. Manso C. F. Guerra Rodrigo F. (1996). Hyaluronic Acid in Progressive Systemic Sclerosis. Dermatology 192, 46–49. 10.1159/000246314 8832952

[B11] Goldin J. G. Lynch D. A. Strollo D. C. Suh R. D. Schraufnagel D. E. Clements P. J. (2008). High-Resolution CT Scan Findings in Patients with Symptomatic Scleroderma-Related Interstitial Lung Disease. Chest 134, 358–367. 10.1378/chest.07-2444 18641099

[B12] Heickendorff L. Zachariae H. Bjerring P. Halkier-Sørensen L. Søndergaard K. (1995). The Use of Serologic Markers for Collagen Synthesis and Degradation in Systemic Sclerosis. J. Am. Acad. Dermatol. 32, 584–588. 10.1016/0190-9622(95)90341-0 7896946

[B13] Hutchinson M. Abignano G. Blagojevic J. Bosello S. L. Allanore Y. Denton C. (2021). Op0269 A Combined Clinical and Biomarker Algorithm to Predict Fvc Decline in Systemic Sclerosis Associated Interstitial Lung Disease: Results from an International Multicentre Observational Cohort. Ann. Rheum. Dis. 80, 2–164. 10.1136/annrheumdis-2021-eular.1861

[B14] Ichikado K. Suga M. Muranaka H. Gushima Y. Miyakawa H. Tsubamoto M. (2006). Prediction of Prognosis for Acute Respiratory Distress Syndrome with Thin-Section CT: Validation in 44 Cases. Radiology 238, 321–329. 10.1148/radiol.2373041515 16293804

[B15] Kikuchi K. Kubo M. Sato S. Fujimoto M. Tamaki K. (1995). Serum Tissue Inhibitor of Metalloproteinases in Patients with Systemic Sclerosis. J. Am. Acad. Dermatol. 33, 973–978. 10.1016/0190-9622(95)90289-9 7490368

[B16] Kikuchi K. Kadono T. Furue M. Tamaki K. (1997). Tissue Inhibitor of Metalloproteinase 1 (TIMP-1) May Be an Autocrine Growth Factor in Scleroderma Fibroblasts. J. Invest. Dermatol. 108, 281–284. 10.1111/1523-1747.ep12286457 9036925

[B17] Lapière C. M. Lenaers A. Kohn L. D. (1971). Procollagen Peptidase: An Enzyme Excising the Coordination Peptides of Procollagen. Proc. Natl. Acad. Sci. U S A. 68, 3054–3058. 10.1073/pnas.68.12.3054 5289249 PMC389589

[B18] LeRoy E. C. (1974). Increased Collagen Synthesis by Scleroderma Skin Fibroblasts *In Vitro*: a Possible Defect in the Regulation or Activation of the Scleroderma Fibroblast. J. Clin. Invest. 54, 880–889. 10.1172/JCI107827 4430718 PMC301627

[B19] Lichtinghagen R. Pietsch D. Bantel H. Manns M. P. Brand K. Bahr M. J. (2013). The Enhanced Liver Fibrosis (ELF) Score: Normal Values, Influence Factors and Proposed Cut-Off Values. J. Hepatol. 59, 236–242. 10.1016/j.jhep.2013.03.016 23523583

[B20] Nagy Z. Czirják L. (2005). Increased Levels of Amino Terminal Propeptide of Type III Procollagen Are an Unfavourable Predictor of Survival in Systemic Sclerosis. Clin. Exp. Rheumatol. 23, 165–172. 15895885

[B21] Nielsen M. J. Nedergaard A. F. Sun S. Veidal S. S. Larsen L. Zheng Q. (2013). The Neo-Epitope Specific PRO-C3 ELISA Measures True Formation of Type III Collagen Associated with Liver and Muscle Parameters. Am. J. Transl. Res. 5, 303–315. 23634241 PMC3633973

[B22] Parkes J. Roderick P. Harris S. Day C. Mutimer D. Collier J. (2010). Enhanced Liver Fibrosis Test Can Predict Clinical Outcomes in Patients with Chronic Liver Disease. Gut 59, 1245–1251. 10.1136/gut.2009.203166 20675693

[B23] Parkes J. Guha I. N. Roderick P. Harris S. Cross R. Manos M. M. (2011). Enhanced Liver Fibrosis (ELF) Test Accurately Identifies Liver Fibrosis in Patients with Chronic Hepatitis C. J. Viral Hepat. 18, 23–31. 10.1111/j.1365-2893.2009.01263.x 20196799

[B24] Plastiras S. C. Karadimitrakis S. P. Kampolis C. Moutsopoulos H. M. Tzelepis G. E. (2007). Determinants of Pulmonary Arterial Hypertension in Scleroderma. Semin. Arthritis Rheum. 36, 392–396. 10.1016/j.semarthrit.2006.10.004 17204309

[B25] Rosenberg W. M. Voelker M. Thiel R. Becka M. Burt A. Schuppan D. (2004). Serum Markers Detect the Presence of Liver Fibrosis: A Cohort Study. Gastroenterology 127, 1704–1713. 10.1053/j.gastro.2004.08.052 15578508

[B26] Scheja A. Akesson A. Hørslev-Petersen K. (1992). Serum Levels of Aminoterminal Type III Procollagen Peptide and Hyaluronan Predict Mortality in Systemic Sclerosis. Scand. J. Rheumatol. 21, 5–9. 10.3109/03009749209095054 1570489

[B27] Scheja A. Wildt M. Wollheim F. A. Akesson A. Saxne T. (2000). Circulating Collagen Metabolites in Systemic Sclerosis. Differences between Limited and Diffuse Form and Relationship with Pulmonary Involvement. Rheumatology (Oxford) 39, 1110–1113. 10.1093/rheumatology/39.10.1110 11035131

[B28] Stone P. J. Korn J. H. North H. Lally E. V. Miller L. C. Tucker L. B. (1995). Cross-linked Elastin and Collagen Degradation Products in the Urine of Patients with Scleroderma. Arthritis Rheum. 38, 517–524. 10.1002/art.1780380409 7718005

[B29] Vali Y. Lee J. Boursier J. Spijker R. Löffler J. Verheij J. (2020). Enhanced Liver Fibrosis Test for the Non-invasive Diagnosis of Fibrosis in Patients with NAFLD: A Systematic Review and Meta-Analysis. J. Hepatol. 73, 252–262. 10.1016/j.jhep.2020.03.036 32275982

[B30] Webber J. Meran S. Steadman R. Phillips A. (2009). Hyaluronan Orchestrates Transforming Growth Factor-beta1-dependent Maintenance of Myofibroblast Phenotype. J. Biol. Chem. 284, 9083–9092. 10.1074/jbc.M806989200 19193641 PMC2666557

[B31] Yoshizaki A. Iwata Y. Komura K. Hara T. Ogawa F. Muroi E. (2008). Clinical Significance of Serum Hyaluronan Levels in Systemic Sclerosis: Association with Disease Severity. J. Rheumatol. 35, 1825–1829. 18688912

[B32] Young-Min S. A. Beeton C. Laughton R. Plumpton T. Bartram S. Murphy G. (2001). Serum TIMP-1, TIMP-2, and MMP-1 in Patients with Systemic Sclerosis, Primary Raynaud's Phenomenon, and in normal Controls. Ann. Rheum. Dis. 60, 846–851. 11502611 PMC1753839

